# More than labels: neural representations of emotion words are widely distributed across the brain

**DOI:** 10.1093/scan/nsae043

**Published:** 2024-06-21

**Authors:** Kent M Lee, Ajay B Satpute

**Affiliations:** Department of Psychology, Northeastern University, 125 Nightingale Hall, Boston, MA 02115, USA; Department of Psychology, Northeastern University, 125 Nightingale Hall, Boston, MA 02115, USA

**Keywords:** emotion words, language, MVPA, representational similarity analysis

## Abstract

Although emotion words such as “anger,” “disgust,” “happiness,” or “pride” are often thought of as mere labels, increasing evidence points to language as being important for emotion perception and experience. Emotion words may be particularly important for facilitating access to the emotion concepts. Indeed, deficits in semantic processing or impaired access to emotion words interfere with emotion perception. Yet, it is unclear what these behavioral findings mean for affective neuroscience. Thus, we examined the brain areas that support processing of emotion words using representational similarity analysis of functional magnetic resonance imaging data (*N* = 25). In the task, participants saw 10 emotion words (e.g. “anger,” “happiness”) while in the scanner. Participants rated each word based on its valence on a continuous scale ranging from 0 (Pleasant/Good) to 1 (Unpleasant/Bad) scale to ensure they were processing the words. Our results revealed that a diverse range of brain areas including prefrontal, midline cortical, and sensorimotor regions contained information about emotion words. Notably, our results overlapped with many regions implicated in decoding emotion experience by prior studies. Our results raise questions about what processes are being supported by these regions during emotion experience.

Words such as “anger” or “fear” are typically viewed as mere labels of the emotions they signify. Yet, accumulating evidence from behavioral and neuropsychology studies suggests that emotion words may play a more fundamental role in constructing emotion representations (Lindquist and Gendron 2013, Lindquist et al. 2015b, 2016). For instance, developmental studies examining the ages at which children can distinguish between facial expression stimuli have found a “verbal superiority effect” (e.g. Russell and Bullock 1985, Widen 2013; see also Hoemann et al. 2019, Nook et al. 2017). What this effect entails is that children are better able to match facial expression stimuli to emotion words (e.g. a scowling face to the word “angry”) than to other similar facial expression stimuli (i.e. another scowling face). Similarly, adults are also faster and more accurate when matching facial expression stimuli with emotion words than with other facial expression stimuli (Nook et al. 2015).

The notion that words are important for emotion representation is bolstered by causal evidence that emotion words can influence emotion perception. For instance, interfering with emotion word processing also interferes with perceiving facial expressions as belonging to particular emotion categories (e.g. by using articulatory suppression, Roberson and Davidoff 2000; or semantic satiation, Gendron et al. 2012, Lindquist et al. 2006; for a review see Fugate 2013). Damage to the brain regions important for semantic processing in cases of semantic aphasia also impairs emotion perception. For example, damage to the anterior temporal lobes (ATLs) produces deficits in verbal tasks such as object labeling and word completion (Mummery et al. 2000, Chan et al. 2001, Snowden et al. 2018) and also results in deficits in emotion perception, even when tasks do not explicitly require using emotion labels (Lindquist et al. 2014, Souter et al. 2021). These findings suggest that emotion words, or more precisely the semantic categories they connote, are commonly accessed even in the routine processing of emotion perceptions.

The implications of this work for the neuroscience of emotion have yet to be fully understood, in part, because little is known regarding which brain regions carry information pertaining to emotion word content. Studies in affective neuroscience have typically focused on examining the brain regions that encode or decode full-fledged experiences (e.g. Sabatinelli et al. 2005, Kassam et al. 2013, Bush et al. 2017) or perceptions (e.g. Adolphs et al. 1999, Kesler et al. 2001, Matsuda et al. 2013, Skerry and Saxe 2014, Liang et al. 2017) of emotion. However, if the semantic concepts connoted by emotion words are routinely retrieved during the experience of emotions, then it stands to reason that at least some of the brain regions that carry representational content for emotional experiences and perceptions may do so because they carry information concerning semantic representations of emotion words.

The findings from prior functional magnetic resonance imaging (fMRI) studies are suggestive of this possibility. Many of the brain regions that are often engaged during the experience and/or perception of emotion (Kober et al. 2008, Vytal and Hamann 2010, Lindquist et al. 2012) have also been implicated in semantic processing (Binder and Desai 2011, Satpute and Lindquist 2021). These regions include the lateral temporal cortex, the ATL, and the ventrolateral (vlPFC) and dorsomedial prefrontal cortices (dmPFC). In prior work, we (Wager et al. 2015, Satpute and Lindquist 2019, 2021) and others (Ochsner and Barrett 2001, Barrett et al. 2007) suggested that the reason why so many widely distributed brain regions are implicated in emotion is because an emotional experience is a construction involving a collection of neurocognitively dissociable psychological processes (for similar ideas from an appraisal perspective, see Brosch and Sander 2013, Smith and Lane 2015, Sander et al. 2018). Consistent with this idea, in previous research we found functional dissociations between the amygdala, which tracked with affective arousal; the dmPFC, which tracked the focus of attention on affective dimensions of experience; and the vlPFC, which tracked semantic categorization into verbal labels (Satpute et al. 2013). While all of these brain regions are often engaged during experiences and perceptions of emotion, they appear to play distinct functional roles when constructing these states. These findings are consistent with the constructionist account that emotion perceptions and experiences involve a wide variety of more basic psychological ingredients (Barrett 2006, 2009, 2017, Lindquist and Barrett 2012, Lindquist 2013).

However, the univariate approach taken in our prior work (Satpute et al. 2013) limits the ways in which content associated with emotion words may be encoded by the brain. That is, while this prior work has identified which brain regions are engaged during the process of semantic categorization in general, it remains unclear whether and which brain regions carry content information that differentiates between emotion word representations. This same issue pertains to studies on “affect labeling,” which have also implicated the vlPFC in the process of categorization (Lieberman et al. 2007, 2011, Brooks et al. 2017), but not the semantic contents of emotion word representations.

Perhaps more pertinent to this research question are studies using multivoxel pattern analysis (MVPA), wherein the patterns of activity across voxels are used to decode certain psychological states or task conditions (Norman et al. 2006, Haxby 2012, Poldrack et al. 2012). MVPA studies in affective neuroscience have focused in particular on decoding specific categories of emotional experiences (Kragel and LaBar 2015, Wager et al. 2015, Saarimäki et al. 2016), emotion perceptions (Ethofer et al. 2009, Peelen et al. 2010, Said et al. 2010), or valence and arousal states (Baucom et al. 2012, Bush et al. 2017, 2018, Kim et al. 2017, Shinkareva et al. 2020). In concert with univariate studies, results from studies using MVPA also suggest that brain regions that carry information about emotion categories overlap with those previously implicated in semantic processing (Satpute and Lindquist 2019, 2021). Even so, these studies typically view representations tied to emotion words as an issue in study design (e.g. Kassam et al. 2013, Saarimäki et al. 2016), rather than an important component of emotion construction (Barrett et al. 2011, Lindquist and Gendron 2013, Lindquist et al. 2015b, Mesquita 2022).

## The current study

Here, we ask which brain regions carry information about emotion word content even in the absence of a full-fledged emotional experience? We used an existing fMRI dataset in which participants were simply shown emotion words (e.g. “Fear,” “Guilt,” “Calm,” “Pride,” etc.) and were instructed to judge each word for its hedonic (pleasant/unpleasant) or evaluative (good/bad) valence properties in alternating blocks. We previously reported which brain regions are differentially associated with hedonic versus evaluative emotion knowledge retrieval by comparing activity during these blocks (Lee et al. 2022). In the current study, we focused instead on which brain regions carry information pertaining to emotion words. Importantly, the simple valence judgments were useful for ensuring that participants accessed the semantic rather than just lexical aspects of words. We then used a type of representational similarity analysis (RSA) to identify which brain regions carry informational content about emotion words.

## Method

### Participants

Twenty-five right-handed, native English speakers (11 females, 14 males) aged 21–40 years (M = 29.84, SD = 5.46) took part in the study. Inclusion criteria for the study were for participants to be right-handed, aged between 18 years and 55 years, native English speakers, have no non-removable metal in their bodies, and no history of psychiatric illness. Our sample size was chosen based on sample sizes of contemporary MVPA studies of emotion, which typically included 21 participants or fewer (Kassam et al. 2013, Shinkareva et al. 2014, Kragel and LaBar 2015, Saarimäki et al. 2016). Prior to participation, all participants provided informed consent, and all procedures were approved by the California Institute of Technology’s institutional review board. We note that these participants’ data were previously published in Lee et al. (2022).

### Experimental design

While undergoing fMRI, participants engaged in an emotion concept valuation task. Specifically, they were shown blocks of emotion words consisting of an equal number of negatively- and positively-valenced emotion concepts: Anger, Disgust, Fear, Guilt, Sad, Calm, Excited, Happy, Lust, and Pride. In each block, each word was presented serially on the screen for 4 s in randomized order with a variable interstimulus interval [2–4 s], and participants were instructed to judge each emotion word on one of two valuation scales. The ratings were made during the 4-s period that the emotion words were on screen. In the hedonic rating blocks, participants were first shown the instruction cue, “Pleasant to Unpleasant” on the screen for 5 s. During the subsequent block of emotion words, participants made pleasantness ratings of the emotion concepts connoted by the emotion words on a continuous scale [0–1] from “Pleasant” to “Unpleasant” using a trackball mouse. In the evaluative rating blocks, participants were instead shown the instruction cue, “Good to Bad” on the screen for 5 s, and made corresponding ratings during the block of emotion words. These valuation judgments were included for two reasons. First, for another study, we were interested in identifying which brain regions were associated with focussing on evaluative versus hedonic emotion knowledge. We reported these findings in another manuscript (Lee et al. 2022). Second, and more relevant for the present purposes, these valence judgments helped to ensure that participants were engaged in the task and were retrieving semantic knowledge about the emotion words.

Participants completed 8 blocks (80 trials, 8 trials/emotion word) of the task, evenly divided into 4 blocks per valuation condition, with blocks randomized across two runs. Finally, before each block, participants completed a standard active baseline task (Stark and Squire 2001); participants were serially presented with eight single-digit numbers [1, 3, 4, 5, 5, 5, 6, 8] in a randomized order for 1.7 s each, and instructed to make a button response if the number was a “5” participants. Participants were trained on the task by completing the equivalent of one run outside of the scanner. For interpretability, we reverse scored the ratings such that unpleasant/bad was coded as 0 and pleasant/good was coded as 1.

### Apparatus

The fMRI was conducted using a 3 T Siemens TIM Trio (Erlangen, Germany) equipped with a 32-channel head coil. Functional images (60 slices) were acquired with a T2*-weighted echo planar imaging pulse sequence (repeitition time [TR] = 1  s, echo time [TE] = 30 ms, flip angle = 60°, 2.5 mm isotropic resolution, interleaved transverse acquisition, multi-band acceleration factor = 4). Structural images were acquired using a T1-weighted sequence (TR = 2.4 s, TE = 2.6 s, flip angle = 8°, 1 mm isotropic resolution). During fMRI, the experimental task was projected onto a screen that participants viewed through a mirror attached to the head coil.

### Data analysis

Preprocessing of functional images was done using the fmriprep pipeline (https://fmriprep.readthedocs.io/en/stable/index.html). Preprocessing included coregistration of functional images with T1-weighted structure images, motion and slice-time correction, and normalization of functional images to the MNI-ICBM152 template. Afterward, functional images were spatially smoothed (6 mm full-width half-max). First-level models were conducted using NeuroElf v1.1 rc2 (http://neuroelf.net/) software on the MATLAB R2018b (Mathworks) platform. A general linear model was used to estimate hemodynamic responses during each emotion word, separately for each run and each participant. Thus, each model included 10 regressors (one for each emotion word) convolved with a canonical hemodynamic response function, along with nuisance regressors for six motion regressors and a high-pass temporal filter (discrete cosine transform, 100 s). Betas from the first-level analyses were exported as NIFTI files.

The relevant scripts for the analyses, behavioral data, and first-level input data for the RSA (see below) are available on the Open Science Foundation website at this link: https://osf.io/wmszj/. Currently, the preprocessed data are not fully in Brain Imaging Data Structure format; however, they are available upon request.

#### Representational similarity analysis

We conducted RSA (Kriegeskorte et al. 2008, Kriegeskorte and Kievit 2013) to identify brain regions that contained information about emotion words. RSA examines how similar (or dissimilar) the pattern of activation across a set of voxels (here, beta-weights from first-level models) is during one condition in comparison to another. We specifically used the CoSMoMVPA toolbox (Oosterhof et al. 2016) in MATLAB to perform our searchlight RSA with the correlation method (Haxby 2012). We first applied a custom mask to the data due to signal dropout. Our custom mask combined the Harvard-Oxford cortical and subcortical atlas (1 mm, 25% probability) and included only voxels where data were present for 60% (*N* = 15) of participants (see https://osf.io/wmszj/).

After applying the mask to the data, we used the CoSMoMVPA toolbox to perform a whole-brain searchlight RSA. The searchlight analysis examined a spherical neighborhood with a radius of five voxels around each voxel. Split-half correlations for the betas were computed for on-diagonal (i.e. within emotion category) and off-diagonal values (i.e. between emotion categories). The difference was then taken between on- and off-diagonal coefficients resulting in a dissimilarity score. Values closer to zero reflect greater similarity while values further away from zero reflect greater dissimilarity.

#### Statistical thresholding of RSA maps

We thresholded the maps of dissimilarity scores via signed permutation testing in MATLAB R2018b with 5000 iterations and *α* = 0.05. Specifically, we create an n-voxel vector of the average similarity score across participants for each voxel. Next, we randomly assigned each voxel to have a negative or positive value. This process was repeated across 5000 iterations, producing a matrix of n-voxels × 5000 iterations that served as our null distribution. We then added the observed data (i.e. mean similarity scores) to this matrix and obtained the cutoff point at the 95th percentile. We then created a thresholded map that masked out all similarity scores that fell below the cutoff.

#### Follow-up analyses

In our follow-up analyses, we tested the robustness of our results and examined whether other features of the emotion words, such as word valence or word length might account for our results. In order to do so, we parcellated the regions identified by our RSA into 27 individual areas shown in prior studies to be associated with affective processing (e.g. orbitofrontal cortex, anterior cingulate cortex, the insula), concept representation (e.g. medial and lateral prefrontal cortices, precuneus, and posterior cingulate cortex), motor behavior (e.g. pre- and postcentral gyri), and visual processing of linguistic stimuli [bilateral lateral occipital cortices (LOCs), the lingual gyrus, and the fusiform gyrus].

Next, we used multiple regression to examine the extent to which factors such as valence and word length accounted for our multivariate results. For each region, in each participant, we estimated a regression equation predicting the beta-weights (activity) during one run in each voxel using the beta-weights from the other run, while controlling for the valence ratings and length of the emotion word. Specifically, we examined within-category activity across runs. Because the evaluative and hedonic valence ratings across all emotion words were highly correlated (*r* = 0.99, *P* < .001) and because differences between the types of ratings were not of interest in the present study, we averaged together both sets of ratings. The purpose of averaging together the evaluative and hedonic ratings was primarily to examine if valence might explain our results. Thus, we used the averaged ratings as the valence rating regressor in our follow-up analyses. All variables were *z*-scored prior to analyses. For each region, we then averaged together the regression coefficients across participants and conducted one-tailed *t*-tests to examine whether those coefficients were significantly different than zero at the group level.

## Results

### Representational similarity

**Figure 1.**
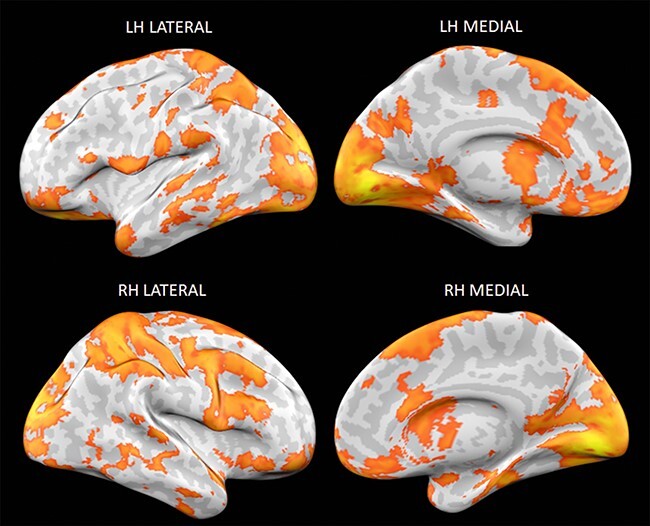
The RSA results. Displayed are areas that contain information about emotion knowledge (*P* < .05). The top row shows the left hemisphere in lateral and medial views while the bottom row shows the right hemispheres in lateral and medial views. LH = left hemisphere, RH = right hemisphere.

**Figure 2.**
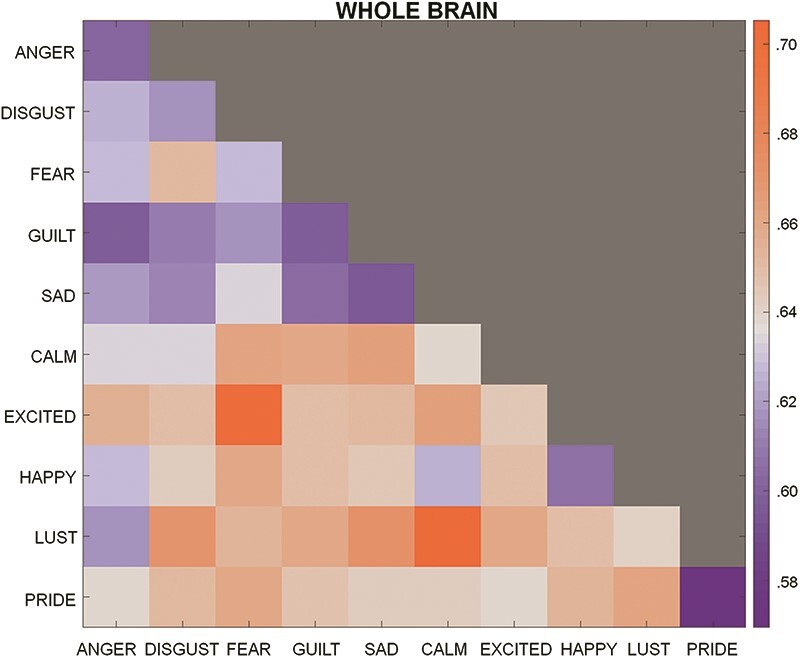
The RDM for whole-brain representational similarity results depicts dissimilarity scores computed by taking the average of the correlation matrix of whole-brain cross-run activity across subjects. We then subtracted the coefficients from two to obtain a dissimilarity score such that higher numbers reflect greater dissimilarity and lower numbers reflect greater similarity. Note: Although there appears to be a difference in similarity between negatively-valenced versus positively-valenced emotions on the diagonals, this effect was not significant, *t* (4) = −1.38, *P* = .24. Orange = more dissimilarity, Blue = more similarity.

Figure 1 displays the thresholded results (*P* < .05) of our whole-brain searchlight RSA. We found a wide range of regions that contained information about emotion knowledge. Our results included areas previously associated with affective experience (e.g. orbitofrontal cortex, anterior cingulate cortex, the insula) and default mode network areas (e.g. medial and lateral prefrontal cortex, precuneus). Our results also included a number of sensorimotor areas including the pre- and postcentral gyri, the superior and inferior parietal lobules, and wide swathes of occipital cortex. Notably, within the occipital cortex, we also found involvement of left and right LOCs in representation of emotion words. Figure 2 depicts the representational dissimilarity matrices (RDMs) for regions of interest of limbic and paralimbic, as well as default mode areas.

### Follow-up analyses

The involvement of regions associated with the generation of affect such as the anterior cingulate cortex and insula raises questions about whether involvement of these regions may be due to the valence of the emotion concepts. Similarly, involvement of motor areas may reflect ratings based on valence, and the involvement of visual regions might be accounted for by differences in the visual features of the stimuli (i.e. word length). Thus, we probed whether valence and word length might account for some of our findings in follow-up analyses using multiple regression. All standardized regression coefficients and *t*-test statistics are displayed in Table 1.

**Table 1.** Results of follow-up analyses using multiple regression predicting neural activity related to emotion words in one run from neural activity related to emotion words in another run, while controlling for valence and word length.

	MNI Coordinates				Emotion			Valence			Word length		
Area	*x*	*y*	*z*	*k*	*β*	*t*	*P*	*β*	*t*	*P*	*β*	*t*	*P*
Emotion and valence													
ACC	0	24	18	94	0.10	2.40	.02	−0.09	−2.97	.01	−0.07	−1.55	.14
SFG	15	−3	75	780	0.24	5.71	<.001	−0.06	−2.12	.04	−0.05	−1.53	.14
L Precentral gyrus	−57	6	9	85	0.20	4.86	<.001	−0.09	−2.52	.02	−0.04	−1.03	.31
R Precentral/IFG	57	6	6	141	0.11	2.48	.02	−0.09	−2.83	.01	−0.02	−0.70	.49
L LOC	−33	−81	−15	313	0.35	6.29	<.001	−0.05	−2.23	.04	−0.03	−0.62	.54
R LOC	18	−81	−12	332	0.29	5.44	<.001	−0.06	−2.81	.01	0.02	0.95	35
Emotion													
OFC	−15	42	−24	171	0.07	2.36	.03	>−0.01	−0.22	.82	<0.01	0.04	.97
L Anterior temporal	−48	9	−30	136	0.13	4.82	<.001	−0.04	−1.19	.25	−0.03	−0.83	.41
L lPFC	−18	42	−18	219	0.10	2.92	.01	−0.04	−1.87	.07	−0.03	−1.59	.13
R lPFC	42	54	−15	180	0.16	3.80	<.001	−0.05	−1.23	.23	−0.04	−1.34	.19
R Anterior temporal	33	18	−42	55	0.05	2.03	.05	−0.02	−0.59	.56	0.02	0.70	.49
R Superior temporal/insula	51	18	−18	160	0.11	2.22	.04	−0.06	−1.74	.09	−0.04	−1.17	.25
R precentral	51	0	54	340	0.20	3.76	<.001	−0.05	−1.73	.10	−0.03	−0.95	.35
L postcentral/parietal lobule	−21	−42	75	643	0.32	6.80	<.001	−0.04	−1.67	.11	−0.01	−0.20	.85
L postcentral/parietal	−66	−18	27	124	0.25	4.92	<.001	−0.07	−1.82	.08	−0.02	−0.49	.63
R postcentral	57	−12	51	173	0.21	4.16	<.001	<0.01	0.07	.95	−0.03	−0.67	.51
R Superior parietal	39	−48	66	1090	0.26	5.29	<.001	−0.04	−1.33	.19	−0.01	−0.25	.81
R MFG	42	27	33	393	0.22	4.33	<.001	−0.02	−0.54	.59	−0.04	−1.12	.27
L MFG	−30	48	36		0.18	4.31	<.001	−0.06	−1.75	.09	−0.03	−0.55	.59
R MTG	69	−27	0	136	0.16	2.88	.01	−0.04	−0.95	.35	−0.02	−0.61	.55
PCC/Cuneus	9	−60	−3	256	0.21	4.93	<.001	−0.02	−0.91	.37	−0.05	−1.29	.21
L STG	−69	−24	6	181	0.24	5.31	<.001	−0.04	−1.17	.25	−0.02	−0.69	.50
Valence													
R IFG/STG	51	21	−18	52	0.06	1.10	.28	−0.11	−2.79	.01	−0.06	−1.17	.25
Neither													
mPFC	9	57	−27	330	0.03	1.36	.19	<0.01	0.18	.86	>−0.01	−0.18	.86
L IFG	−24	21	−24	105	0.03	0.79	.44	>−0.01	−0.12	.91	−0.02	−0.92	.37
L Precuneus	−6	−66	36	67	0.08	1.66	.11	−0.06	−1.39	.18	<0.01	0.01	.99
Visual	3	−75	−12	3443	0.05	1.39	.18	−0.01	−1.96	.06	−0.01	−0.94	.36

Separate multiple regression equations were estimated for each region of interest. Regions under the category “Emotion and Valence” reflect regions in which activity related to emotion words were uniquely predicted by emotion words and valence even after controlling for word length. Regions under the category “Emotion” reflect regions in which only emotion words contributed unique variance (i.e. showed only representational similarity) after controlling for valence and word length. Regions labeled “Valence” reflect areas in which activity during viewing of emotion words was uniquely predicted only by valence while controlling for emotion words and word length. Finally, regions under “Neither” are regions in which representational similarity no longer held after controlling for valence and word length, and was not predicted by valence or word length. L = left hemisphere, R = right hemisphere, ACC = Anterior Cingulate Cortex, PCC = Posterior Cingulate Cortex, STG = Superior Temporal Gyrus, mPFC = Medial Prefrontal Cortex, OFC = Orbital Prefrontal Gyrus, lPFC = Lateral Prefrontal Cortex, MFG = Middle Frontal Gyrus, SFG = Superior Frontal Gyrus, MTG = Middle Temporal Gyrus, IFG = Inferior Temporal Gyrus, MNI = Montreal Neurologic Institute.

**Figure 3.**
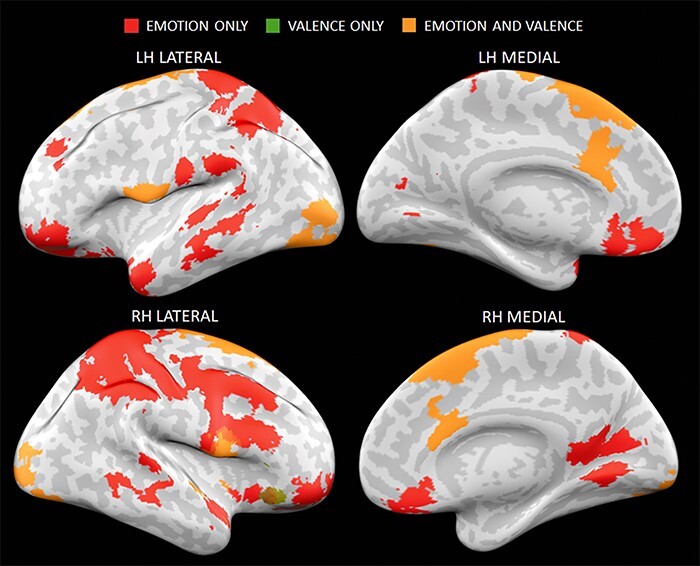
Regions that continued to contain information about emotion words even after controlling for hedonic valence and word length (Red). Figure also displays regions in which activity related to emotion words was uniquely predicted by emotion words and valence (Orange). One cluster that included the right inferior frontal gyrus and superior frontal gyrus was uniquely predicted by only valence (Green; bottom left figure). Lateral and medial views of the left hemisphere are displayed in the top row, while lateral and medial views of the right hemisphere are displayed in the bottom row. LH = left hemisphere, RH = right hemisphere.

Even after controlling for valence and word length, we continued to find that 21 out of 27 regions contained information about emotion words (see Table 1 and Fig. 3). Of the regions that showed continued representational similarity for emotion words, some areas also showed sensitivity to valence (orange in Fig. 3) while others only showed sensitivity to emotion words (red in Fig. 3). Almost all of the areas that showed sensitivity to valence above and beyond emotion words and word length overlapped with regions that showed continued representational similarity to emotion words. The one exception was a cluster that included the right inferior frontal gyrus and superior temporal gyrus (green in Fig. 3). Finally, no regions showed sensitivity to word length above and beyond emotion words or valence.

## Discussion

Increasing evidence points to language as playing an integral role in emotion representation (Lindquist et al. 2015a, Brooks et al. 2017, Shablack and Lindquist 2019, Satpute and Lindquist 2021). Using words such as “anger” or “fear” to convey feelings is not just descriptive of mental experience. Rather, words and language, more broadly, may influence how people develop emotion category representations (Nook et al. 2017, Hoemann et al. 2019, 2020, Shablack et al. 2020). In turn, these representations influence how they construct perceptions (Roberson and Davidoff 2000, Lindquist et al. 2006, Gendron et al. 2012) and experiences (Lindquist and Barrett 2008, Oosterwijk et al. 2010, Lee et al. 2018) of emotion. Yet, the implications of these behavioral findings for the neural representation of emotion have remained unclear.

Thus, in order to extend the behavioral work on language and emotion to affective neuroscience, we investigated the brain regions that may carry information pertaining to emotion concepts when simply processing emotion words. In our study, participants made simple semantic judgments regarding the valence of certain emotion words in the complete absence of evocative stimulus presentations. A RSA showed that brain regions containing information pertaining to emotion words were widely distributed across limbic and paralimbic regions, prefrontal and midline cortical areas, and sensorimotor areas even after controlling for valence and word length. Our findings suggest that the mere retrieval of emotion knowledge from emotion words can lead to widely distributed patterns of activation throughout much of the brain.

Our study lies at the intersection of two disparate research areas in affective neuroscience: one on the neuroscience of language and emotion, the other on the neural representation of discrete emotions. The first area has focused on the neurocognitive processes involved in putting feelings into words, such as identifying which brain regions underlie attending to and labeling feelings (e.g. Burklund et al. 2014, Lieberman et al. 2007, Satpute et al. 2013;Taylor et al. 2003). Data are typically averaged across emotion categories in univariate analyses to reveal brain regions that support verbal categorization, introspection, etc. The second research area has focused on identifying distinct patterns of activation that occur between experiences of emotion (e.g. Kassam et al. 2013, Kragel and LaBar 2015, Wager et al. 2015, Saarimäki et al. 2016). Participants are induced to feel various emotions using evocative stimuli, and MVPA is used to identify which brain regions carry information that may distinguish between experiences from distinct emotion categories. These research areas have largely developed independently of one another. Here, our study serves as a bridge that may open up new directions in understanding the neural basis of both putting feelings into words, and how emotion words may contribute to emotion category representations. Below, we discuss the implications of our findings for these two areas in turn, and we further comment on the implications of these findings for emotion theory and for individual differences in language and emotion processing, such as in alexithymia.

### Putting feelings into words

The ability to communicate feelings with words may provide unique mechanistic pathways to process and regulate feelings (Greenberg 2011, Lieberman et al. 2011, Niles et al. 2015, Torre and Lieberman 2018, Nook et al. 2021). In part for these reasons, one branch of research in affective neuroscience has focused on the neural systems that underlie the ability to attend to and label or categorize perceptions and experiences of emotion (Lane et al. 1997, Morawetz et al. 2017, Torre and Lieberman 2018, Satpute and Lindquist 2021). However, most of this work has focused on the cognitive processes involved in matching or labeling emotion words with perceptions or feelings of emotion irrespective of the particular contents of specific emotion words. For example, studies typically compare trials in which affective faces are matched to emotion word labels versus various control conditions, irrespective of whether those trials involve different emotion category content (e.g. Lieberman et al. 2007). These task-level effects are typically subjected to univariate analyses and tend to produce more focal activation patterns in particular brain regions, such as the ventrolateral prefrontal cortex (Satpute et al. 2013).

In contrast to those studies, we used MVPA to identify brain regions that carry informational content in their distribution of functional activation patterns pertaining to specific emotion words. In doing so, we found that information about emotion content was widely distributed throughout much of the brain. These findings complement prior work on affect labeling and emotion categorization by illustrating how conceptual content for specific emotion words, too, may be an important factor for developing a complete understanding on the neuroscience of language and emotion (also see, Satpute et al. 2016). Of interest to future work is understanding how certain cognitive processes (e.g. demands placed on the retrieval and selection of particular emotion words) relate with the neural representation of specific emotion concepts.

### Implications for studies on the neural signature of emotion

Several prior studies have shown that the information contained in patterns of activity across the brain can be used to decode emotional categories (Kassam et al. 2013, Kragel and LaBar 2015, Wager et al. 2015, Saarimäki et al. 2016). Some of these studies have claimed to identify “neural signatures” of emotion, implying that a given emotion category has a specific, monolithic activation pattern that underlies instances from the category. However, we and others have shown that the psychological meaning of these patterns of activity is quite ambiguous (Clark-Polner et al. 2016). For instance, in prior work, we found that the overwhelming majority of fear-predictive patterns of activity are actually content dependent; different brain regions predict increasing degrees of fear depending on whether the situation involves heights or spiders (Wang et al. 2022; see McVeigh et al. 2024 for a similar argument concerning peripheral autonomic predictors of fear). In our other work, we have argued that these results suggest that fear does not involve a single brain pattern or state. Rather, fear may instead involve a collection of brain states that depend on the person and situation.

Even though participants in our study were not induced to experience emotions, our findings (see Figs 1 and 3) bear a striking resemblance to those reported in prior MVPA studies of emotional experience (as reviewed in Kragel and LaBar 2016, Nummenmaa and Saarimäki 2019, Satpute and Lindquist 2019, 2021) that used highly evocative affective stimuli. To be sure, an ideal study would directly compare the extent to which patterns of activation during full-fledged emotional experiences are shared from the mere processing of emotion words to determine whether and which brain regions do contain shared neural codes. While our study is limited in this respect, there are a few noteworthy observations. In both cases, patterns of activity carrying information pertaining to emotion categories are widely distributed throughout the brain and also appear to involve many of the same brain regions. Such an overlap raises important issues concerning the degree to which the neural codes underlying the classification of emotion pertain to affective aspects of the emotional experience versus other components including those evoked by emotion word representations. In that regard, our findings dovetail with recent work showing people can readily indicate pleasure or displeasure based on relatively unemotional, semantic associations (Itkes et al. 2017, Itkes and Kron 2019, Hamzani et al. 2020). Prior MVPA studies did not include conditions that compared decoding accuracy for relatively shallow/conceptual representations of emotion versus deeply felt instances of emotion, which would be an important avenue to pursue going forward.

### The role of language in emotion representation

Our findings also have theoretical implications regarding the role of emotion words in the neural basis of emotion representation. By some accounts, one might expect that only “higher-level,” multimodal association areas would carry information about emotion categories when accessed from semantic processing of emotion words. Certain lines of work in the neuroscience of language have pointed to the multimodal association areas (e.g. lateral temporal cortex, anterior medial, and ventrolateral prefrontal cortex), as being involved in the semantic processes more generally (Binder et al. 2009). However, our findings showed that primary somatosensory areas also carried information that distinguished between emotion words. These early sensory areas have also been theoretically hypothesized to contain information pertaining to “felt” emotions (Damasio and Carvalho 2013).

Our findings are consistent with certain constructionist theories that argue emotion words play a constitutive role in the construction of emotion (Lindquist et al. 2015a, Brooks et al. 2017, Satpute and Lindquist 2019, 2021). By this account, emotion words serve to catalyze the formation of emotion concept representations (e.g. during childhood development). Consistent with this idea, the ability of young children to differentiate different sensory stimuli (e.g. emotional faces) into distinct emotion categories above and beyond valence coincides closely with the development of language (Widen 2016, Nook et al. 2017, Hoemann et al. 2019, 2020). Conversely, the absence of emotion word usage during development can also interfere with formation of discrete emotion representations (as in alexithymia or “affective agnosia”; Taylor et al. 1999, Taylor and Bagby 2000, Lane et al. 2015).

The notion that language plays an important role in concept formation is not unique to emotion. In cognitive psychology, verbal labels catalyze the formation of concept representations in object perception, too, even when words are incidental to the task at hand (Lupyan et al. 2007). Aligning with constructionist theories in emotion, grounded and embodiment theories of semantic representations in cognitive psychology suggest that concept representations of words are “grounded” or derive their meaning from the sensory-motor representations of the objects themselves (Barsalou 1999, 2008, Gallese and Lakoff 2005, Wilson-Mendenhall et al. 2013, Mazzuca et al. 2021). For instance, when participants are asked to confirm whether or not a noun (e.g. “tomato”) can be described by features such as shape (e.g. roundness) and color (e.g. red), this engages sensorimotor regions involved in shape and color perception (Fernandino et al. 2016). It follows suit that the meanings of emotion words like “anger,” “fear,” “happy,” and “pride” are also grounded in the instances wherein those words were used, such that mere reading of the word involves pattern completion of the aspects of the affective instances themselves. From this view, the distinctions between emotion concepts and emotional experiences are not necessarily one of kind (cf. Adolphs 2017), but rather reflect an artificial boundary (Hoemann and Feldman Barrett 2019), wherein pattern completion leads to some amount of overlapping patterns of activity whether originating from reading emotion words or being presented with evocative stimuli.

### Alexithymia and the neural representations of emotion words

Our findings also raise new questions and research directions concerning individual differences in the ability to put feelings into words (Taylor and Bagby 2000, Tugade et al. 2004, Lane et al. 2020, Hoemann et al. 2021). Many people find it difficult to understand and describe their experiences in terms of emotions. This trait has been most commonly referred to as alexithymia (Sifneos 1996), albeit it has also been linked to the closely related constructs of affective agnosia (Lane et al. 2020) and emotion granularity (Tugade et al. 2004; for a conceptual framework on emotion expertise, see Hoemann et al. 2021). Here, we use the term alexithymia for brevity in our discussion, but we note that the implications may extend to these overlapping constructs as well.

Alexithymia has been linked with a wide variety of clinical conditions and an inability to reap the benefits of psychotherapy (Sifneos 1996, Taylor et al. 1999). Several potential mechanisms may explain alexithymia in part depending on the level of analysis (e.g. sociocultural, developmental, or neurocognitive; Taylor et al. 1999). Focusing on the neurocognitive level, it is possible that high alexithymia is associated with general processes pertaining to the retrieval of emotion words, regardless of the specific content of those emotion words. By this processing account, certain processes (e.g. affect labeling and emotion categorization) as studied in previous work may underlie individual differences in alexithymia. Another (not mutually exclusive) possibility is that high alexithymia is associated with degraded representational content (i.e. low precision) for emotion words. By this representational account, we might expect that neural variation that accounts for individual differences in alexithymia would be explained by measures extracted from MVPA of specific emotion words using a design similar to the present study.

### Limitations

The results of our study should be understood in the context of its limitations. One limitation of our study is that we did not include a condition that induced full-fledged emotion experiences in participants. In future studies, a direct comparison between neural activity during processing of emotion words and full-blown emotion experiences may elucidate why there is an overlap between regions involved in processing emotion words and regions previously implicated in induced emotion experiences.

Another limitation of our study is that we did not include ratings of arousal for the emotion words. It is possible that arousal may account for neural representations of emotion words showing greater within-category similarity versus cross-category similarity. For instance, neural activity related to emotion words associated with high arousal might be similar to other emotion words with high arousal. This would lead to results showing greater similarity along the diagonal of the RDMs on average. However, we note that although we were unable to statistically control for arousal, the results of the whole-brain RDM (Fig. 2) do not seem consistent with this arousal account. For example, the arousal account would expect “anger” to show greater similarity to “excited” given that they are often thought of as high arousal emotions. Conversely, we might expect “sad” to show greater similarity to “calm” given that they are often considered to be low arousal emotions. Yet, we did not find this to be the case. Nevertheless, future studies would benefit from including ratings of arousal alongside ratings of valence to account for the possibility that these broader affective factors influence neural representations of emotion words. Moreover, including ratings of valence and arousal may be critical for studies that seek to understand the neural representations of full-blown emotion experiences, as our results overlap with regions identified in prior studies of induced emotion (e.g. Sabatinelli et al. 2005, Kassam et al. 2013, Bush et al. 2017).

## Summary and conclusion

We found that emotion words engaged a diverse collection of areas in the brain. These areas ranged from sensorimotor regions (e.g. occipital areas, pre- and postcentral gyri, the insula) associated with experience to multimodal areas (e.g. the lateral and medial prefrontal cortex, the orbitofrontal gyrus) thought to integrate sensory signals with concept knowledge. These findings raise questions about the extent to which brain areas that represent emotion knowledge and full-blown emotion experiences overlap, and why. Rather than being simple semantic labels, our results are consistent with the idea that language, and words in particular, may play a more integral role in constructing emotion experience.
